# Postoperative systemic inflammation after major abdominal surgery: patient‐centred outcomes

**DOI:** 10.1111/anae.16104

**Published:** 2023-08-02

**Authors:** C. R. Bain, P. S. Myles, C. Martin, S. Wallace, M. A. Shulman, T. Corcoran, R. Bellomo, P. Peyton, D. A. Story, K. Leslie, A. Forbes, the RELIEF trial investigators, Paul Myles, Rinaldo Bellomo, Tomas Corcoran, Chris Christophi, Andrew Forbes, Phil Peyton, David Story, Kate Leslie, Jonathan Serpell, Shay McGuinness, Rachel Parke, Sophie Wallace, M Mythen, R Gruen, J McNeil, G Ludbrook, K Lee, P Peyton, A Forbes, S Wallace, A Meehan, D McIlroy, M Shulman, DJ Cooper, PS Myles, S Wallace, C Farrington, A Ditoro, W Galagher, M Pollock, A Neylan, R Bellomo, P Peyton, D Story, S Sidiropoulos, S Baulch, A Carter, S Jacups, JL Reynolds, J Rowley A J Neal, E J Bendall, J R Sutherland, R Bulach, A Wang, NLT Tan, C Osborne, A Marriott, K Ives, B Wakefield, A Quail, J Douglas, I Boden, D Blackford, A Chuan, I Seppelt, A Wu, B Rodriguez, L Siu, R Robinson, L Bulfin, A A Beck, V Wilkinson, B Riedel, A Melville, U Gurunathan, M Bennett, A Duggan, P Sivalingam, B Moser, T Bott, S Sawhney, M Duroux, T Painter, M Chapman, J Moore, S Lang, J Hayer, R Koronis, N Terblanche, D Cooper, R Turner, R Seale, M Challis, K Gard, K Leslie, R Cotter, T Corcoran, TD Phan, P Corcoran, Y Uda, V Nguyen, D Bramley, AM Southcott, J Grant, H Taylor, S Bates, M Towns, A Tippett, F Marshall, J van Vlymen, M Jaeger, D DuMerton Shore, H Sato, T Sato, V Chan, R JinSA McCluskey, N Ayach, MTV Chan, PWY Chiu, WWK Wu, M Tsang, G Landoni, R Lembo, S McGuinness, R Parke, F Pugh, D McAllister, P Dalley, S Reddy, E Ridgeon, S Hurford, L Navarra, R Sol Cruz, G Minto, S Harwood, A Patrick, A Pai, A Kaliappan, M Vertue, J Sonksen, R Gidda, A Chishti, C Scott, S Jakkampudi, P Watt, Z Milan, S Birch, G Kunst, D Martin, S James, M Pinto, RCF Sinclair, C Scott, A Addei, S Cope, H Melsom, L Duncan, A Kurz, D Sessler, S Miller, M Brawley, KO Pryor, SC Marcott, LA Pharmer

**Affiliations:** ^1^ Department of Anaesthesiology and Peri‐operative Medicine Alfred Hospital and Monash University Melbourne VIC Australia; ^2^ Department of Anaesthesiology and Peri‐operative Medicine Alfred Hospital and Monash University Melbourne VIC Australia; ^3^ School of Public Health and Preventive Medicine Monash University Melbourne VIC Australia; ^4^ Department of Anaesthesiology and Peri‐operative Medicine Alfred Hospital and Monash University Melbourne VIC Australia; ^5^ Department of Anaesthesiology and Peri‐operative Medicine Alfred Hospital and Monash University Melbourne VIC Australia; ^6^ Department of Anaesthesia and Pain Medicine Royal Perth Hospital Perth WA Australia; ^7^ Department of Critical Care University of Melbourne Melbourne VIC Australia; ^8^ Australian and New Zealand Intensive Care Research Centre Monash University Melbourne VIC Australia; ^9^ Department of Anaesthesia Austin Hospital Heidelberg VIC Australia; ^10^ Department of Critical Care University of Melbourne Melbourne VIC Australia; ^11^ Department of Anaesthesia and Pain Management Royal Melbourne Hospital Melbourne VIC Australia; ^12^ School of Public Health and Preventive Medicine Monash University Melbourne VIC Australia

**Keywords:** anaesthesia, disability‐free survival, major surgery, patient‐reported outcomes, quality of recovery, systemic inflammation

## Abstract

Postoperative systemic inflammation is strongly associated with surgical outcomes, but its relationship with patient‐centred outcomes is largely unknown. Detection of excessive inflammation and patient and surgical factors associated with adverse patient‐centred outcomes should inform preventative treatment options to be evaluated in clinical trials and current clinical care. This retrospective cohort study analysed prospectively collected data from 3000 high‐risk, elective, major abdominal surgery patients in the restrictive vs. liberal fluid therapy for major abdominal surgery (RELIEF) trial from 47 centres in seven countries from May 2013 to September 2016. The co‐primary endpoints were persistent disability or death up to 90 days after surgery, and quality of recovery using a 15‐item quality of recovery score at days 3 and 30. Secondary endpoints included: 90‐day and 1‐year all‐cause mortality; septic complications; acute kidney injury; unplanned admission to intensive care/high dependency unit; and total intensive care unit and hospital stays. Patients were assigned into quartiles of maximum postoperative C‐reactive protein concentration up to day 3, after multiple imputations of missing values. The lowest (reference) group, quartile 1, C‐reactive protein ≤ 85 mg.l^‐1^, was compared with three inflammation groups: quartile 2 > 85 mg.l^‐1^ to 140 mg.l^‐1^; quartile 3 > 140 mg.l^‐1^ to 200 mg.l^‐1^; and quartile 4 > 200 mg.l^‐1^ to 587 mg.l^‐1^. Greater postoperative systemic inflammation had a higher adjusted risk ratio (95%CI) of persistent disability or death up to 90 days after surgery, quartile 4 vs. quartile 1 being 1.76 (1.31–2.36), p < 0.001. Increased inflammation was associated with increasing decline in risk‐adjusted estimated medians (95%CI) for quality of recovery, the quartile 4 to quartile 1 difference being ‐14.4 (‐17.38 to ‐10.71), p < 0.001 on day 3, and ‐5.94 (‐8.92 to ‐2.95), p < 0.001 on day 30. Marked postoperative systemic inflammation was associated with increased risk of complications, poor quality of recovery and persistent disability or death up to 90 days after surgery.

## Introduction

Complications following major surgery contribute to an estimated eight million deaths annually [1, 2]. The impact of peri‐operative inflammation and immune function on surgical outcomes is now, more than ever, appreciated following the findings of global surgical collaboration into the timing of surgery after SARS‐CoV‐2 infection [3, 4]. Inflammation after major abdominal surgery is essential and balanced by pro‐ and anti‐inflammatory processes within the innate and adaptive immune systems, stimulating natural repair and healing [5, 6, 7]. However, dysregulated hyperinflammation and/or immunosuppression may increase the risk of postoperative complications and organ dysfunction and contribute to poorer quality of recovery, persistent disability, or death [2, 8, 9, 10]. Most analyses to date use plasma C‐reactive protein (CRP) concentrations [11, 12] to assess the impact of different levels of inflammation on short‐term surgical and infectious complications [13, 14, 15] and longer‐term overall survival [16, 17, 18]. The consequences of higher levels of postoperative systemic inflammation on patient‐centred outcomes are unclear [19, 20].

Accordingly, we investigated the relationship between postoperative systemic inflammation and quality of recovery, disability and complications following major abdominal surgery. Our primary hypothesis was that adults with higher levels of postoperative systemic inflammation had a higher rate of complications, poorer quality of recovery and poor disability‐free survival following major abdominal surgery when compared with patients with lower levels of postoperative systemic inflammation.

## Methods

This retrospective cohort study adheres to the Strengthening The Reporting of Observational Studies in Epidemiology (STROBE) statement [21].

We compared patients with different levels of systemic inflammation based on postoperative CRP concentrations. The study data were prospectively collected from the restrictive versus liberal fluid therapy for major abdominal surgery (RELIEF) trial [22], a large pragmatic, multicentre, randomised trial in which patients having major abdominal surgery were assigned randomly to either a restrictive (zero balance) or liberal intravenous fluid regimens. Patients were stratified by site and planned high dependency or intensive care unit (HDU/ICU) admission. Briefly, patients in the liberal group received an initial intravenous fluid bolus of 10 ml.kg^‐1^ of balanced salt solution (Hartmann's) followed by 8 ml.kg^‐1^.h^‐1^ until the end of surgery, and maintenance of 1.5 ml.kg^‐1^.h^‐1^ for 24 h. The zero balance‐restrictive group received approximately half this intravenous fluid volume [22].

Our inclusion criteria, as defined by the RELIEF trial [22], were patients undergoing elective major abdominal surgery at high risk of postoperative complications. The exclusion criteria were: patients undergoing non‐elective or time‐critical surgery; ASA physical status 5; liver resections; and minor surgery such as laparoscopic cholecystectomy or hernia repair. Patient allocations were blinded and further details are available elsewhere [22]. Ethics committee approval was obtained at all sites before the commencement of this cohort study and all patients provided informed consent.

Enhanced recovery after surgery care principles [23] were recommended and all patients received prophylactic antibiotics according to established guidelines. Medications were mostly continued peri‐operatively but withholding ACE inhibitors and angiotensin receptor‐blocking drugs on the day of surgery was recommended. The use of pre‐operative bowel preparation, fasting times, enhanced recovery after surgery data, medications (including steroids), biochemistry and haematology results were recorded on the case report form. Anaesthetic drugs and peri‐operative analgesia were left to the discretion of the anaesthetists. These data were recorded. In addition, low intra‐operative and recovery room blood pressure, defined as a systolic blood pressure < 90 mmHg [24] for more than 5 min requiring treatment (either additional intravenous fluid or vasopressor therapy) was also recorded.

Patients were followed daily, and outcomes were recorded until discharge. Serum electrolytes, albumin, haemoglobin and 12‐lead ECG were ordered pre‐operatively and on postoperative day 1. On postoperative day 3, all patients completed a 15‐item quality of recovery score (QoR‐15) [25]. C‐reactive protein was measured on postoperative day 3, and additional tests were ordered when indicated, including additional CRP levels whenever infection or sepsis was suspected. We recommended that antihypertensive medications be withheld until the patients' blood pressures were consistently at pre‐operative levels.

On day 30, all patients were contacted by telephone to ascertain if they had experienced any of the study outcomes and, if detected, further testing was arranged. The QoR‐15 was repeated on day 30 along with the World Health Organisation Disability Assessment Schedule 2.0 (WHODAS) and the WHODAS was repeated at 3, 6 and 12‐month follow‐up to ascertain survival status and the onset of new disability [26].

Our co‐primary endpoint was persistent disability or death up to 90 days after surgery and the QoR‐15 score [25] at days 3 and 30. Persistent disability was defined as a WHODAS 2.0 score of at least 24/48 points at days 30 and 90 [26]. This represents a disability level of at least 25% and is the threshold between disabled and not disabled according to the WHO guidelines [27]. Disability was recorded by the patients. However, if they were unable to report reliably, we used a proxy's assessment.

Secondary endpoints included: 90‐day and 1‐year all‐cause mortality; septic complications (a composite of sepsis, surgical site infection, anastomotic leak and pneumonia); acute kidney injury; unplanned admission to ICU; total ICU stay; hospital length of stay; and hospital readmission at 3, 6 and 12 months. Each endpoint has had pre‐specified definitions reported previously [22]. Sepsis and surgical site infection were assessed according to the Center for Disease Control and Prevention and National Healthcare Safety Network surveillance definitions. Pneumonia was defined as the presence of new or progressive pulmonary infiltrates on the chest radiograph and two or more of fever (≥38.5 °C or postoperative hypothermia < 36 °C); leucocytosis (≥12,000 white blood cell.ml^‐1^) or leukopenia (<4000 white blood cell.ml^‐1^); purulent sputum and/or worsening cough or dyspnoea. Anastomotic leak was defined as a defect in the intestinal wall at the anastomotic site leading to communication between intra‐ and extraluminal compartments. Acute kidney injury was defined according to the Kidney Disease Improving Global Outcomes (KIDGO) Group criteria, excluding urine output. Stage 2 or worse acute kidney injury was defined as at least two‐fold increase in creatinine or estimated glomerular filtration rate decrease by 50% [28].

All outcomes were clearly defined in the trial protocol and the outcome data were collected by research staff blinded to treatment group allocation. Most outcomes were confirmed by independent blinded assessors on the endpoint adjudication committee from the original source documentation. To better describe the potential relationships between confounding factors, mediating factors and outcomes we include a direct acyclic graph (online Supporting Information Figure S1).

The sample size calculation was based primarily on our own data and other published studies [29, 30]. This resulted in a final sample size of 3000 that provided 80% power for the original RELIEF trial [22].

All statistical analyses were performed with the intention‐to‐treat population of the RELIEF trial [22] but with its own pre‐specified statistical analysis plan (online Supporting Information Appendix S2). Increasing levels of postoperative systemic inflammation were categorised into quartiles based on maximum postoperative CRP concentrations up to day 3, hereafter titled ‘inflammation groups’, with the lowest quartile being the reference group. Descriptive statistics were used to compare the baseline characteristics of patients in the inflammation groups. For patients missing all postoperative CRP measurements to day 3, their maximum CRP concentration to day 3 was imputed using multiple imputations with chained equations, including baseline and post‐baseline variables (up until postoperative day 3) predictive of CRP measurements being missing, and including the relevant outcome variables in the imputation models. Each imputed maximum CRP to day 3 measurement was categorised and imputed into one of the inflammation group quartiles. A total of 20 imputed datasets were produced, with results combined across imputations using Rubin's rules for each of the analyses specified below.

The co‐primary outcome, persistent disability or death up to 90 days, was compared between inflammation groups using log‐binomial regression to estimate risk ratios (RRs) and 95%CI. The QoR‐15 scores at days 3 and 30 were compared between inflammation groups using median regression to produce estimated differences between medians with 95%CIs. A sensitivity analysis was used to adjust for: RELIEF randomised group; age; sex; ASA physical status; smoker status; Charlson comorbidity index score; pre‐operative steroid use; surgical technique (open, laparoscopic, conversion to open from laparoscopic); planned ICU/HDU admission; duration of surgery; surgery for cancer; blood transfusion up to postoperative day 3; blood loss; and pre‐operative white cell count.

The secondary outcomes, 90‐day mortality; major septic complications (composite of sepsis, surgical site infection, pneumonia, anastomotic leak); acute kidney injury; unplanned ICU admission; and hospital readmissions, were compared between inflammation groups using log‐binomial regression to estimate RRs and 95%CIs directly. Duration of stay outcomes was compared across inflammation groups using parametric accelerated failure time models, with a log‐normal distribution, yielding estimated ratios of median durations with 95%CI. To depict both the full and independent effect of inflammation, unadjusted and adjusted analyses were completed as previously described.

An exploratory subgroup analysis was done for persistent disability or death to 90 days, acute kidney injury, surgical site infection, hospital length of stay and postoperative day 3 QoR‐15 for each CRP inflammation group by RELIEF trial randomised group. We undertook tests for interaction by adding treatment‐by‐endpoint terms to the regression models specified for the main analyses of each outcome (online Supporting Information Table S1).

We also conducted an additional unadjusted analysis of other reported biomarkers of inflammation (lowest albumin up to day 3, highest white cell count to discharge, highest temperature to discharge). We used median regression to compare these biomarkers for the inflammation groups to the reference group (online Supporting Information Table S2).

## Results

Study patients were enrolled in the RELIEF trial between May 2013 and September 2016. For the present analysis, data on the primary and secondary outcomes were available for 2983 (mean [SD] age, 66 [13] y, 1429 [52.1%] men) at 47 centres in seven countries. Patient and peri‐operative characteristics of patients who had at least one CRP concentration measured to day 3 postoperatively (n = 2533) are presented in Table 1. The lowest quartile (Q1, reference group, n = 639) had a maximum CRP up to day 3 ≤ 85 mg.l^‐1^ while the highest inflammation group (Q4, n = 618) had maximum CRP up to day 3 > 200 mg.l^‐1^, with a peak of 587 mg.l^‐1^.

**Table 1:** Patient and peri‐operative characteristics according to maximum postoperative CRP quartiles up to day 3. Values are mean (SD), number (proportion) or median (IQR [range]).

Factor (CRP mg.l^‐1^)	q1: ≤85	q2: >85–140	q3: >140–200	q4: >200–587
n*	639	634	642	618
Age	62.5 (14.4)	67.8 (12.1)	68.6 (11.8)	67.5 (11.4)
Male	261 (40.8%)	338 (53.3%)	361 (56.2%)	366 (59.2%)
Country				
Australia	327 (51.2%)	347 (54.7%)	340 (53.0%)	408 (66.0%)
Canada	131 (20.5%)	106 (16.7%)	141 (22.0%)	36 (5.8%)
Other	181 (28.3%)	181 (28.5%)	161 (25.1%)	174 (28.2%)
Body weight; kg	86.0 (68.0–113.0 [38.0–237.0])	82.0 (67.3–99.7 [37.0–191.5])	80.0 (67.1–98.0 [36.3–230.0])	82.6 (69.0–98.5 [42.0–236.0])
BMI; kg.m^2^	31.1 (25.1–40.1 [16.1–71.1])	28.7 (24.6–35.0 [15.2–67.9])	28.6 (24.2–34.3 [16.2–74.6])	29.2 (25.4–34.8 [17.0–70.5])
Pre‐operative WHODAS score	15 (13–20 [12–53])	15 (13–20 [12–48])	15 (13–21 [12–49])	16 (13–22 [12–50])
Hypertension	343 (53.7%)	391 (61.7%)	415 (64.6%)	377 (61.0%)
Coronary artery disease	76 (11.9%)	76 (12.0%)	114 (17.8%)	116 (18.8%)
Heart failure	19 (3.0%)	17 (2.7%)	25 (3.9%)	25 (4.0%)
Previous myocardial infarction	38 (5.9%)	41 (6.5%)	68 (10.6%)	70 (11.3%)
Peripheral vascular disease	24 (3.8%)	34 (5.4%)	47 (7.3%)	53 (8.6%)
Charlson comorbidity index score	2 (1–3 [0–12])	2 (2–4 [0–11])	3 (2–4 [0–12])	3 (2–4 [0–10])
Cancer	363 (56.8%)	470 (74.1%)	497 (77.4%)	448 (72.5%)
Current smoker	68 (10.6%)	73 (11.5%)	86 (13.4%)	98 (15.9%)
History of stroke or TIA	21 (3.3%)	55 (8.7%)	58 (9.0%)	57 (9.2%)
COPD	85 (13.3%)	91 (14.4%)	114 (17.8%)	132 (21.4%)
Moderate/severe renal disease	18 (2.8%)	44 (6.9%)	67 (10.4%)	55 (8.9%)
ASA physical status				
ASA 1 or 2	281 (44.0%)	269 (42.4%)	204 (31.8%)	228 (36.9%)
ASA 3 or 4	358 (56.0%)	365 (57.6%)	438 (68.2%)	390 (63.1%)
Aspirin	105 (16.4%)	129 (20.3%)	138 (21.5%)	119 (19.3%)
Pre‐operative steroids	21 (3.3%)	32 (5.0%)	41 (6.4%)	32 (5.2%)
NSAIDS	39 (6.1%)	31 (4.9%)	34 (5.3%)	32 (5.2%)
Baseline haemoglobin; g.l^‐1^	135 (123–145 [83–194])	133 (120–144 [79–182])	132 (118–144 [77–179])	131 (118–143 [73–189])
White cell count; 10^9^.l^‐1^	7.2 (5.9–8.8 [2.4–16.5])	7.3 (5.9–8.9 [2.0–18.8])	7.4 (6.1–8.9 [1.9–33.0])	7.6 (6.2–9.3 [2.3–22.9])
Albumin; g.l^‐1^	39.0 (36.0–42.0 [3.6–66.0])	39.0 (35.0–41.0 [3.0–52.0])	38.0 (35.0–41.0 [3.6–48.0])	38.0 (34.0–41.0 [3.3–49.0])
Proposed surgery				
Gastrointestinal surgery	453 (70.9%)	432 (68.1%)	472 (73.5%)	468 (75.7%)
Renal/urological, gynaecological or other	186 (29.1%)	202 (31.9%)	170 (26.5%)	150 (24.3%)
Surgical technique				
Laparoscopic	333 (52.1%)	212 (33.4%)	128 (19.9%)	91 (14.7%)
Open (includes conversion from laparoscopy)	306 (47.9%)	422 (66.6%)	514 (80.1%)	527 (85.3%)
Cancer surgery	331 (51.8%)	452 (71.3%)	461 (71.8%)	411 (66.5%)
Duration of surgery; h	2.8 (2.1–3.5 [0.6–12.0])	3.2 (2.4–4.2 [0.5–14.8])	3.7 (2.5–5.0 [0.7–11.7])	4.0 (3.0–5.2 [0.3–13.4])
Planned destination ICU/HDU vs. ward	103 (16.1%)	154 (24.3%)	238 (37.1%)	261 (42.2%)
Blood loss; ml	100 (50–250 [0–8000])	200 (100–400 [0–6000])	250 (100–500 [0–5700])	300 (150–500 [0–11,000])
Blood transfusion pre day 3	32 (5.0%)	58 (9.1%)	82 (12.8%)	103 (16.7%)
Discharge alive pre day 3	117 (18.3%)	25 (3.9%)	12 (1.9%)	3 (0.5%)
RCT liberal fluid group	317 (49.6%)	331 (52.2%)	304 (47.4%)	312 (50.5%)

WHODAS, World Health Organisation Disability Assessment Schedule; TIA, transient ischemic attack; COPD, chronic obstructive pulmonary disease; NSAIDS, non‐steroidal anti‐inflammatory drugs; RCT, randomised controlled trial.

* n = 2533 patients with at least one post‐operative CRP to day 3 measurement available.

Patients in the inflammation groups tended to be older, male, have more comorbidities and required cancer surgery. There was a greater need for open surgery, of longer duration, and planned postoperative admission to ICU or HDU. However, baseline haemoglobin, white cell count and albumin concentration were similar between all groups. Furthermore, there was no significant difference in randomised assignment to the restrictive fluid group between the groups (49.6% compared with 52.2%).

A total of 450 patients (15.1%) had missing CRP data. These patients were more likely to be younger and be discharged before day 3, and less likely to be having gastrointestinal or cancer surgery with planned admission to HDU/ICU. However, there were only slightly lower rates of open surgery or higher rates of laparoscopic surgery, and the duration of surgery and comorbidities were similar to those patients where CRP was available (online Supporting Information Table S3).

From the analysis imputing missing CRP values, we observed an increasing proportion of patients with persistent disability or death up to 90 days in all inflammation groups compared with the reference group (Q1:10.8% vs. Q2:13.2%, Q3:18.2% vs. Q4:25.6%). Compared with the reference group, the unadjusted RR (95%CI) was higher when the maximum postoperative day 3 CRP was > 140 mg.l^‐1^, Q3 vs. Q1 RR 1.67 (1.27–2.22), p < 0.001, and continued to rise as the max CRP increased to > 200 mg.l^‐1^, Q4 vs. Q1 RR 2.36 (1.81–3.08) p < 0.001. After RR adjustment (95%CI) a maximum postoperative day 3 CRP > 200 mg.l^‐1^ remained significantly associated with an increased risk of persistent disability or death, Q4 vs. Q1 RR 1.76 (1.31–2.36) p < 0.001 (Table 2).

**Table 2:** Primary and secondary outcomes according to multiply imputed CRP quartiles (n = 2983). Quality of recovery values are estimated 50th (25th and 75th) percentiles, their test is differences in medians (95%CI); length of stay values are estimated 50th (25th and 75th) percentiles, their test is ratio of medians (95%CI); the remainder are estimated proportions, test is risk ratio.

Factor (CRP; mg.l^‐1^)	Q1	Q2	Q3	Q4	Unadjusted				Adjusted			
					Q2 vs. Q1	Q3 vs. Q1	Q4 vs. Q1	Equality of RR/medians test	Q2 vs. Q1	Q3 vs. Q1	Q4 vs. Q1	Equality of RR/medians test
	CRP ≤ 85	CRP > 85–140	CRP > 140–200	CRP > 200–587								
Primary outcomes												
Disability to 90 days or death	10.8%	13.2%	18.2%	25.6%	1.22 (0.89–1.66), p = 0.21	1.67 (1.27–2.22), p < 0.001	2.36 (1.81–3.08), p < 0.001	p < 0.001	1.08 (0.78–1.49), p = 0.63	1.29 (0.95–1.74), p = 0.10	1.76 (1.31–2.36), p < 0.001	p < 0.001
Quality of recovery, day 3	116.9 (102.1–129.0)	109.5 (91.8–123.2)	103.1 (87.1–116.8)	97.4 (78.2–112.7)	‐7.45 (‐10.50 to ‐4.40), p < 0.001	‐13.80 (‐16.73 to ‐10.87), p < 0.001	‐19.50 (‐22.85 to ‐16.15), p < 0.001	p < 0.001	‐5.26 (‐8.30 to ‐2.22), p < 0.001	‐9.27 (‐12.42 to ‐6.13), p < 0.001	‐14.04 (‐17.38 to ‐10.71), p < 0.001	p < 0.001
Quality of recovery, day 30	135.1 (120.0–144.2)	132.3 (116.8–142.4)	130.3 (113.8–140.0)	124.7 (107.0–137.8)	‐2.85 (‐5.31, ‐0.39), p = 0.02	‐4.75 (‐7.37 to ‐2.13), p < 0.001	‐10.40 (‐13.87 to ‐6.93), p < 0.001	p < 0.001	‐1.85 (‐4.16–0.46), p = 0.12	‐1.84 (‐4.12–0.44), p = 0.11	‐5.94 (‐8.92 to ‐2.95), p < 0.001	p = 0.002
Secondary outcomes												
Mortality at 90 days	1.3%	0.9%	1.4%	3.1%	0.69 (0.23–2.07), p = 0.51	1.14 (0.43–2.99), p = 0.79	2.51 (1.09–5.79), p = 0.03	p = 0.03	0.43 (0.14–1.33), p = 0.14	0.56 (0.19–1.60), p = 0.28	1.34 (0.51–3.51), p = 0.55	p = 0.05
Mortality at 12 months	4.4%	5.2%	7.7%	9.0%	1.17 (0.73–1.88), p = 0.52	1.73 (1.09–2.73), p = 0.02	2.03 (1.31–3.17), p = 0.002	p = 0.004	0.72 (0.45–1.15), p = 0.17	0.78 (0.49–1.25), p = 0.31	0.97 (0.61–1.54), p = 0.88	p = 0.33
Septic complications imputed composite at 30 days (confirmed)	9.1%	14.4%	25.5%	34.4%	1.58 (1.16–2.14), p = 0.003	2.80 (2.14–3.64), p < 0.001	3.78 (2.92–4.89), p < 0.001	p < 0.001	1.44 (1.05–1.97), p = 0.02	2.28 (1.71–3.04), p < 0.001	3.07 (2.3–4.08), p < 0.001	p < 0.001
Acute kidney injury	2.9%	4.6%	5.9%	11.4%	1.58 (0.88–2.83), p = 0.12	2.06 (1.19–3.55), p = 0.010	3.94 (2.40–6.45), p < 0.001	p < 0.001	1.40 (0.76–2.58), p = 0.28	1.60 (0.88–2.89), p = 0.12	2.90 (1.68–4.98), p < 0.001	p < 0.001
Unplanned ICU admission	5.9%	8.4%	11.1%	16.0%	1.41 (0.95–2.11), p = 0.09	1.88 (1.29–2.73), p < 0.001	2.69 (1.91–3.80), p < 0.001	p < 0.001	1.19 (0.79–1.80), p = 0.40	1.40 (0.94–2.09), p = 0.09	2.00 (1.37–2.91), p < 0.001	p < 0.001
ICU/HDU length of stay (censored 30 days)	1.1 (0.9–2.0)	1.3 (1.0–2.8)	1.7 (0.9, 2.9)	1.9 (1.0, 4.1)	1.26 (1.03–1.56), p = 0.03	1.36 (1.13–1.63), p = 0.001	1.74 (1.45–2.09), p < 0.001	p < 0.001	1.15 (0.92–1.42), p = 0.21	1.14 (0.94–1.38), p = 0.19	1.46 (1.21–1.76), p < 0.001	p < 0.001
Hospital length of stay to 30 days with deaths censored	3.6 (2.4, 5.6)	5.5 (3.5, 8.6)	6.7 (4.6, 12.1)	9.5 (6.4, 16.2)	1.51 (1.39–1.65), p < 0.001	2.00 (1.83–2.18), p < 0.001	2.75 (2.53–2.99), p < 0.001	p < 0.001	1.26 (1.16–1.36), p < 0.001	1.46 (1.35–1.58), p < 0.001	1.90 (1.75–2.07), p < 0.001	p < 0.001
Hospital readmission within 3 months	16.8%	20.2%	24.4%	29.9%	1.21 (0.95–1.53), p = 0.12	1.46 (1.18–1.81), p < 0.001	1.79 (1.45–2.21), p < 0.001	p < 0.001	1.13 (0.88–1.44), p = 0.35	1.24 (0.98–1.57), p = 0.07	1.50 (1.18–1.89), p < 0.001	p = 0.004
Hospital readmission within 6 months	26.1%	30.6%	33.1%	40.7%	1.17 (0.98–1.40), p = 0.07	1.27 (1.08–1.50), p = 0.005	1.56 (1.33–1.83), p < 0.001	p < 0.001	1.08 (0.90–1.29), p = 0.43	1.07 (0.89–1.29), p = 0.45	1.31 (1.09–1.56), p = 0.003	p = 0.009
Hospital readmission within 12 months	36.9%	42.9%	46.3%	54.5%	1.16 (1.02–1.33), p = 0.03	1.25 (1.10–1.43), p < 0.001	1.48 (1.31–1.67), p < 0.001	p < 0.001	1.05 (0.91–1.20), p = 0.54	1.07 (0.93–1.23), p = 0.33	1.26 (1.10–1.44), p < 0.001	p = 0.002

Variables adjusted for study treatment, age, gender, ASA status, smoker status, Charlson comorbidity index score, steroid use pre‐surgery, surgery technique (open, laparoscopic, conversion to open from laparoscopic), planned ICU/HDU admission, duration of surgery, surgery for cancer, blood transfusion up to day 3, blood loss and preoperative white cell count.

**Figure 1:**
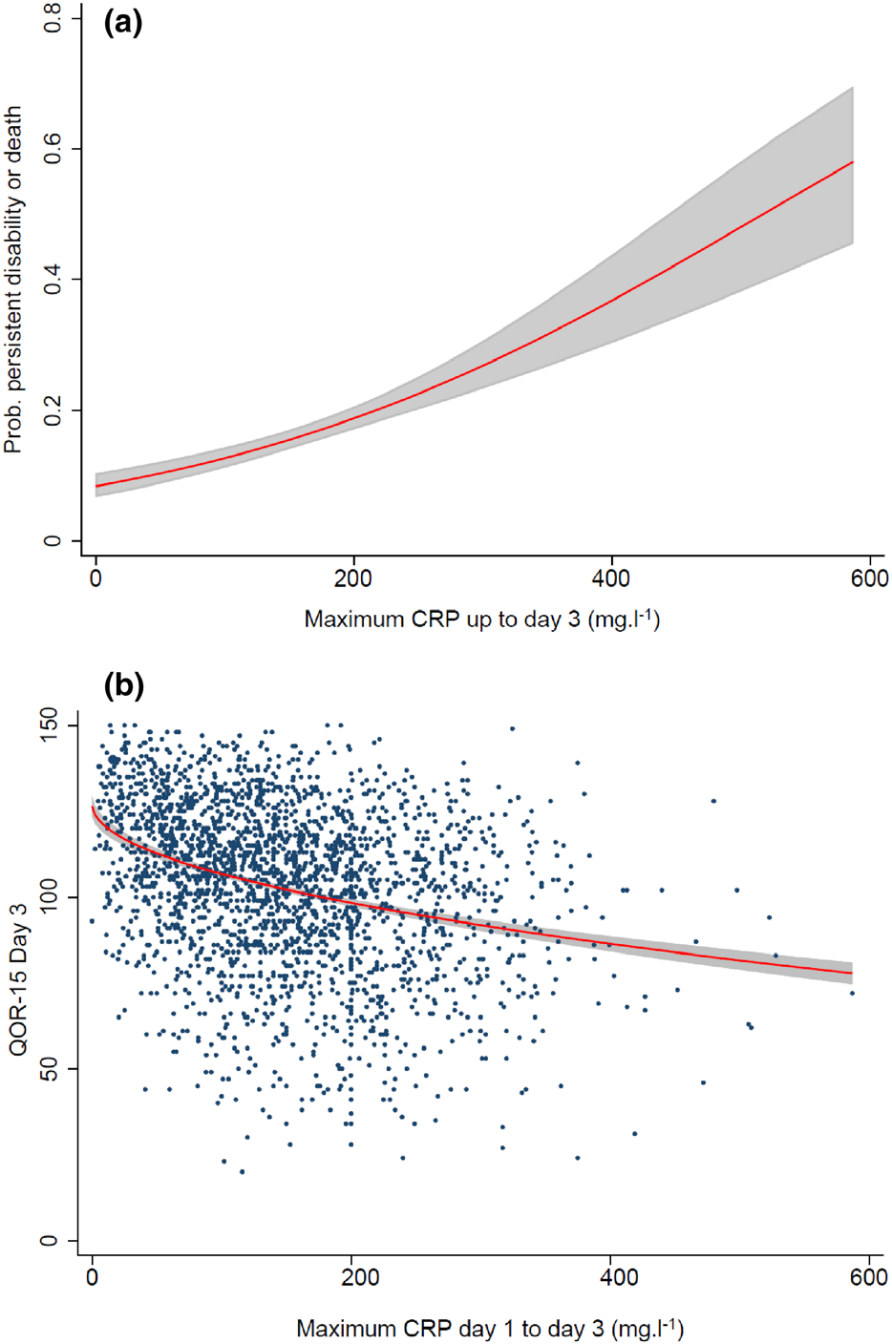
(a) The relationship between measured maximum postoperative C‐reactive protein (CRP) concentration (mg.l^‐1^) up to day 3 and the probability of persistent disability or death up to day 90 after major abdominal surgery; (b) the relationship between maximum postoperative day CRP concentration (mg.l^‐1^) up to day 3 and quality of recovery on day 3. The shaded bands are 95%CI.

Figure 1a demonstrates the positive association between increasing levels of postoperative systemic inflammation (maximum CRP day 1 to day 3) and the probability of persistent disability or death. Above 200 mg.l^‐1^, a rise of 100 mg.l^‐1^, was associated with an increase in the probability of persistent disability or death of approximately 0.1. Therefore, a maximum CRP concentration up to day 3 of 600 mg.l^‐1^ was associated with a probability of persistent disability or death approaching 60%.

We observed an increasing decline in the estimated medians of quality of recovery on day 3 (Q1:116.9, Q2: 109.5, Q3:103.1, Q4:97.4) and day 30 (Q1:135.1, Q2:132.3, Q3:130.3, Q4:124.7) with increasing CRP levels. After adjustment, compared with the 85 mg.l^‐1^ reference level, the estimated medians (95%CI) for quality of recovery on day 3 decreased across the CRP quartiles, Q2 vs. Q1 difference ‐5.26 (‐8.3 to ‐2.22) p < 0.001, Q3 vs. Q1–9.27 (‐12.42 to ‐6.13) p < 0.001, Q4 vs. Q1–14.4 (‐17.38 to ‐10.71) p < 0.001. Patients with postoperative maximum CRP up to day 3 > 200 mg.l^‐1^ also demonstrated lower estimated adjusted quality of recovery on day 30, lower Q4 vs. Q1 difference ‐5.94 (‐8.92 to ‐2.95) p < 0.001, Table 2. Figure 1b demonstrates the unadjusted significant negative association between increasing levels of postoperative systemic inflammation (maximum CRP day 1 to day 3) and quality of recovery on day 3. Small differences in the level of postoperative systemic inflammation, even in the most frequently reported range of 100–200 mg.l^‐1^, were associated with significantly reduced quality of recovery on day 3.

Patients with increased levels of postoperative systemic inflammation had an increasing adjusted RR (95%CI) of septic complications at day 30 (composite including sepsis, surgical site infection, anastomotic leak and pneumonia), Q4 vs. Q1 RR 3.07 (2.31–4.08) p < 0.001; acute kidney injury, Q4 vs. Q1 RR 2.90 (1.68–4.98) p < 0.001; unplanned ICU admission, Q4 vs. Q1 RR 2.0 (1.37–2.91) p < 0.001; ICU/HDU length of stay, Q4 vs. Q1 ratio of medians 1.46 (1.21–1.6) p < 0.001; prolonged hospital length of stay, Q4 vs. Q1 ratio of medians 1.9 (1.75–2.0) p < 0.001; and unplanned hospital readmission within 3 months, Q4 vs. Q1 RR 1.5 (1.18–1.89) p < 0.001; 6 months, Q4 vs. Q1 RR 1.31 (1.09–1.56) p = 0.03; and 12 months RR 1.25 (1.1–1.44) p < 0.001. There was no difference between the inflammation groups and the reference group for mortality at day 90 and 12 months (Table 2).

There were no significant subgroup effects of fluid administration (restrictive/liberal) or the different inflammation groups (Q2–Q4) on persistent disability or death to 90 days, acute kidney injury, surgical site infection, hospital length of stay to 30 days or quality of recovery on day 3. The complete case subgroup analyses are reported in the online Supporting Information Table S2.

Our analysis of the association between maximum postoperative CRP up to day 3 and the other measured inflammatory biomarkers, lowest albumin, highest white cell count to discharge and highest temperature to discharge is presented in online Supporting Information Table S3. All inflammatory groups demonstrated levels consistent with differing degrees of increasing systemic inflammation when compared with the reference group. This finding provides support for the measurement of maximum postoperative CRP up to day 3 as a marker of inflammation.

## Discussion

We have demonstrated a significant association between higher levels of postoperative systemic inflammation, as measured by maximum postoperative CRP up to day 3, with serious complications, poorer quality of recovery and persistent disability or death up to 90 days after major abdominal surgery.

The postoperative stress response includes neurohumoral and inflammatory‐immune components that are determined by the magnitude of surgical injury [11, 31], modified by age [32], co‐existing medical conditions [33, 34] and anaesthesia (immune modulation) [35, 36]. Postoperative CRP concentrations reflect the level of release of cytokines (e.g. interleukin 6) and chemokines in response to tissue injury. C‐reactive protein levels have been shown to reliably reflect the magnitude of surgical injury and are lower after minimally invasive/laparoscopic surgery [11]. Numerous systematic reviews of retrospective analyses in colorectal surgery have highlighted postoperative day‐3 CRP > 150 mg.l^‐1^ to be associated with infectious complications and poorer overall survival [14, 15, 16, 17, 18].

A prospective analysis in 350 patients following major abdominal surgery demonstrated a median (IQR) postoperative day‐3 CRP concentration of 265 (178–324) mg.l^‐1^ in patients with major infectious complications (n = 71, 20.3%). The probability of major infection was < 0.1 when postoperative day‐3 CRP was ≤ 100 mg.l^‐1^ and approximately 0.9 for ≥ 500 mg.l^‐1^ [37]. High postoperative day‐3 CRP also predicts a high postoperative comprehensive complication index [38], a continuous scale of the Clavien‐Dindo classification of surgical morbidity and mortality [39].

Our findings add to these data by highlighting the significant adjusted association between postoperative systemic inflammation and patient‐centred outcomes. Furthermore, by adjusting for confounding factors, our data suggests the CRP levels in Q4 (maximum up to day‐3 CRP > 200 mg.l^‐1^) reflect a level of persisting systemic inflammation (hyperinflammation and immunosuppression) and associated complications (sepsis, surgical site infection, pneumonia, anastomotic leak, acute kidney injury), which may be increasingly mediating a poor quality of recovery, persistent disability or death (Fig. 1). Similarly, the low levels in Q1 (reference level, maximum up to day‐3 CRP ≤ 85 mg.l^‐1^) reflects a resolving host response, promoting wound healing and tissue repair.

When and how the systemic inflammatory‐immune response to surgical injury contributes to patient harm remains an important question. Numerous analyses have demonstrated the utility of low postoperative CRP (<100 mg.l^‐1^) concentrations to support safe early discharge from the hospital [12, 13, 37, 40, 41]. Our findings reinforce this and suggest that the range from 100 mg.l^‐1^ to 200 mg.l^‐1^ represents a ‘transitional zone’ in which caution should be applied as the direction of the change in host response cannot be predicted. However, we posit that, following major abdominal surgery, patients with a postoperative day‐3 CRP > 200 mg.l^‐1^ are experiencing a degree of harmful postoperative systemic inflammatory dysregulation with an increased risk of complications, poorer quality of recovery and persistent disability or death (Fig. 1).

Other than minimally invasive/laparoscopic surgery, enhanced recovery after surgery protocols [42, 43] do not specifically outline steps to limit excessive inflammation [44]. Our data support the measurement of CRP to monitor the magnitude of postoperative systemic inflammation following major abdominal surgery. When elevated, regardless of the surgical approach [40], consideration should be given to potential contributing factors. Future research is justified that focuses on patient‐centred outcomes coupled with analyses at a genomic (epigenome and transcriptome) [45] and functional level (immune cell phenotypes) [46] to discover specific (personalised) changes within the immune system potentially mediating the host response (online Supporting Information Figure S1). Such discovery may facilitate improved prediction [47] and understanding of how immune modifying interventions [5, 48] may be utilised to better target patients at high peri‐operative risk.

The key strengths of this study include the large, multicentre, prospectively collected trial dataset that included extensive peri‐operative variables and patient‐centred outcomes up to one year after surgery. We accounted for confounding variables using multivariable adjustment, sensitivity and subgroup analyses.

Our study has several limitations. First, we did not measure baseline CRP concentrations. Given that surgery was completely elective and other measured baseline markers of inflammation were normal, it is reasonable to assume that the pre‐operative CRP concentrations were also within normal limits. The internal validation of the inflammation groups with the other measured postoperative markers of systemic inflammation also provides separate assurance of the clinical validity of the postoperative CRP measurements utilised in this study. Similarly, we did not measure CRP beyond day 3, and cannot directly compare our findings with studies that measure CRP at days 4, 5 and 6. Second, numerous peri‐operative factors are associated with both the level of postoperative systemic inflammation and poor outcomes. Key factors were, as much as possible, accounted for in our multivariable and subgroup analyses, but residual confounding can still be present that could account for the observed association, for example the use of regional anaesthesia. Third, approximately 15% of patients did not have their CRP measured. As previously described, these patients were more likely to be younger and have less extensive surgery but they were otherwise similar to the patients included in the imputation analysis. Finally, the RELIEF trial was powered to investigate the impact of restrictive vs. liberal fluid administration and was not designed to specifically investigate postoperative systemic inflammation. However, this analysis is the largest, international multicentre observational study to date describing the association between postoperative systemic inflammation, as measured by CRP, to patient‐centred outcomes after major abdominal surgery. Nevertheless, high levels of postoperative day‐3 CRP are significantly associated with adverse postoperative outcomes, and a postoperative CRP > 200 mg.l^‐1^ was associated with an increased risk of serious complications, poorer quality of recovery and persistent disability or death up to 90 days after surgery. Prediction, early detection, and focused treatment strategies based on early CRP measurement may be an important step towards improved patient monitoring and treatment.

## Supporting information


**Appendix S1.** Investigators and committees in the RELIEF Trial.
**Appendix S2.** Statistical analysis plan.

**Figure S1.** Direct acyclic graph outlining the relationship of plasma CRP, postoperative systemic inflammatory dysregulation, primary patient‐centred and other (secondary) outcomes.

**Table S1.** Subgroup analysis for interaction between treatment allocation and outcome in different CRP quartiles.
**Table S2.** Values of other inflammatory markers according to CRP quartiles.
**Table S3.** Patient characteristics by missing maximum CRP up to day 3 divided by missing status.
