# Supplementary material for: Postoperative systemic inflammation after major abdominal surgery: patient‐centred outcomes
Source: Anaesthesia. 2023 Aug 2;78(11):1365–75. doi: 10.1111/anae.16104 (PMC10952313; doi:10.1111/anae.16104)
Supplement: Supplementary file 1 — Appendix S1. Investigators and committees in the RELIEF Trial. Appendix S2. Statistical analysis plan. [file ANAE-78-1365-s001.docx]

**Appendix S1 Investigators and committees in the RELIEF Trial**

***Steering Committee:*** Paul Myles (chair), Rinaldo Bellomo, Tomas Corcoran, Chris Christophi, Andrew Forbes, Phil Peyton, David Story, Kate Leslie, Jonathan Serpell, Shay McGuinness, Rachel Parke, Sophie Wallace

***Data Safety Monitoring Board:*** M Mythen (Chair), R Gruen, J McNeil, G Ludbrook, K Lee

***Data Quality Committee:*** P Peyton, A Forbes, S Wallace, A Meehan

***Endpoint adjudication committee:*** D McIlroy, M Shulman, DJ Cooper

***Funding:*** National Health and Medical Research Council of Australia; New Zealand Health Research Council, Australian and New Zealand College of Anaesthetists.

***Study Sponsor:*** Alfred Health

***UK Sponsor:*** Plymouth NHS trust

**List of principal investigators and study coordinators**

**Australia**

Alfred Hospital: PS Myles, S Wallace, C Farrington, A Ditoro, W Galagher, M Pollock, A Neylan; Austin Health: R Bellomo, P Peyton, D Story, S Sidiropoulos, S Baulch; Cairns Hospital: A Carter, S Jacups; Coffs Harbour Health Campus: JL Reynolds, J Rowley A J Neal, E J Bendall, J R Sutherland; Dandenong Hospital: R Bulach, A Wang; Epworth HealthCare: NLT Tan; Geelong Hospital: C Osborne, A Marriott, K Ives, B Wakefield; John Hunter Hospital; A Quail, J Douglas; Launceston General Hospital: I Boden, D Blackford; Macquarie University Hospital: A Chuan, I Seppelt; Maroondah Hospital: A Wu; Monash Medical Centre: B Rodriguez, L Siu, R Robinson, L Bulfin; Nepean Hospital: A A Beck , V Wilkinson, Peter MacCallum Cancer Centre: B Riedel, A Melville; Prince Charles Hospital: U Gurunathan; Prince of Wales: M Bennett , A Duggan; Princess Alexandra Hospital: P Sivalingam, B Moser, T Bott; Redcliffe Hospital: S Sawhney, M Duroux; Royal Adelaide Hospital: T Painter, M Chapman, J Moore, S Lang, J Hayer, R Koronis; Royal Hobart Hospital: N Terblanche, D Cooper, R Turner, R Seale, M Challis, K Gard; Royal Melbourne Hospital: K Leslie, R Cotter; Royal Perth Hospital: T Corcoran; St.Vincent’s Hospital: TD Phan, P Corcoran, Y Uda, V Nguyen; Western Health: D Bramley, AM Southcott, J Grant, H Taylor, S Bates, M Towns, A Tippett, F Marshall.

**Canada**

Kingston General Hospital, Queen’s University: J van Vlymen, M Jaeger, D DuMerton Shore; Royal Victoria Hospital, McGill University; H Sato, T Sato; University Health Network, Toronto Western Hospital Division: V Chan, R Jin; Toronto General Hospital Division: SA McCluskey, N Ayach.

**Hong Kong**

Prince of Wales: MTV Chan, PWY Chiu, WWK Wu, M Tsang.

**Italy**

San Raffaele Scientific Institute: G Landoni, R Lembo.

**New Zealand**

Auckland CVICU: S McGuinness, R Parke; Auckland Hospital: F Pugh, D McAllister; Wellington Hospital: P Dalley, S Reddy, E Ridgeon, S Hurford, L Navarra, R Sol Cruz.

**United Kingdom**

Plymouth NHS Trust: G Minto (UK Lead), S Harwood (UK administrator), A Patrick; Bassildon and Thurrock: A Pai, A Kaliappan, M Vertue; Dudley Group NHS Foundation Trust; J Sonksen, R Gidda; Freeman Hospital: A Chishti, C Scott; Kettering General Hospital: S Jakkampudi , P Watt; Kings College Hospital NHS Foundation Trust: Z Milan, S Birch, G Kunst; Royal Free Hospital: D Martin, S James, M Pinto; Royal Victoria Infirmary: RCF Sinclair, C Scott; St Georges Healthcare NHS Trust: A Addei; City Hospitals Sunderland NHS foundation Trust: S Cope; University Hospital of North Durham: H Melsom, L Duncan.

**United States of America**

Cleveland Clinic: A Kurz, D Sessler; Wake Forest Baptist Medical Center: S Miller, M Brawley; Weill Medical College of Cornell University: KO Pryor, SC Marcott, LA Pharmer

**Appendix S2 Statistical Analysis Plan**

22 July 2022

**BACKGROUND**

The postoperative stress response includes neurohumoral and inflammatory components and is largely determined by the magnitude of surgical injury. Abdominal surgery elicits a significant response as it breaches protective barriers exposing the immune system to pathogens and cellular debris. Imbalances resulting in hyperinflammation and altered immune competence may increase the risk of postoperative complications and organ dysfunction and hence poorer quality of recovery, persistent disability, or death.

The RELIEF (restrictive versus liberal fluid therapy for major abdominal surgery) trial, published in the New England Journal of Medicine^1^ was a large, definitive randomized trial comparing a restrictive with a more traditional (liberal) fluid regimen in patients undergoing abdominal surgery. Its primary outcome was 1-year disability-free survival.

We propose a substudy of the RELIF trial to investigate the relationship between the postoperative C-reactive protein (CRP) concentrations and septic complications, and both early quality of recovery and 1-year disability-free survival.

STUDY AIMS AND HYPOTHESES

**Aim:** To investigate the relationship between postoperative inflammation and complications, quality of recovery and patient-centred outcomes after major abdominal surgery.

**Hypothesis:** Adults with higher levels of postoperative systemic inflammation, as measured by plasma CRP concentration, have an increased risk of complications and poorer quality of recovery leading to poor disability-free survival following major abdominal surgery when compared with patients with low levels of postoperative systemic inflammation.

**STUDY DESIGN**

A retrospective analysis comparing patients with different levels of postoperative systemic inflammation, using prospective CRP data collected in a large, pragmatic, multicentre, randomized trial in which patients were randomly assigned to either a restrictive (zero balance) or liberal IV fluid regimen, stratified by site and planned HDU/ICU admission.

Increasing levels of postoperative systemic inflammation will be categorised into quartiles based on the post-operative day 3 (POD3) CRP concentrations.

A secondary analysis of POD3 levels up to 100 mg/L (considered as normal, or healthy response) as a reference group will be done.

**ENDPOINT DEFINITIONS**

**Primary Endpoints**

Our co-primary endpoints are:

1. Persistent disability or death by 90 days, where persistent disability was defined as a WHODAS 2.0 score of at least 24 points (on the 48-point scale) at both 30 days and 90 days post-operatively,^26^ reflecting a disability level of at least 25% and being the threshold point between “disabled” and “not disabled” as per WHO guidelines.^29^ Disability was assessed by the participant, but if unable then we used the proxy’s report.
2. Quality of recovery: QoR-15 score^25^ on POD3 and POD30.

**Secondary Endpoints**

Our secondary endpoints are:

1. Death: all-cause mortality at 90 days, then up to 12 months after surgery
2. A composite (pooled) and individual septic complications: sepsis, surgical site infection, anastomotic leak, and pneumonia
   1. Sepsis: using Centers for Disease Control and Prevention (CDC) with National Healthcare Safety Network (NHSN) criteria.^30^
   2. Surgical site infection: using CDC criteria.^30^
   3. Pneumonia: The presence of new and/or progressive pulmonary infiltrates on chest radiograph plus two or more of the following:
      - 1. Fever ≥ 38.5°C or postoperative hypothermia **<**36°C
        2. Leucocytosis ≥ 12,000 WBC/mm^31^ or leucopenia < 4,000 WBC/mm.^31^
        3. Purulent sputum and/or
        4. New onset or worsening cough or dyspnoea.
   4. Anastomotic leak: A defect of the intestinal wall at the anastomotic site (including suture and staple lines of neorectal reservoirs) leading to a communication between the intra- an extra luminal compartments.
3. Acute kidney injury: according to The Kidney Disease: Improving Global Outcomes (KDIGO) group criteria, but not urine output – for Stage 2 or worse AKI defined as at least 2-fold increase in creatinine, or estimated GFR decrease >50%.^32^
4. Unplanned admission to ICU within 30 days of surgery
5. Total ICU stay: additive, including initial ICU admission and readmission times up to POD30
6. Hospital stay: additive, from the start (date, time) of surgery until actual hospital discharge, plus readmission(s) up to POD30
7. Hospital re-admission at 3, 6 and 12 months.

**STATISTICAL ANALYSES**

All statistical analysis will be performed with the intention-to-treat population of the RELIEF trial.[^5^](#_ENREF_5)

Descriptive statistics will be used to compare the baseline characteristics of patients with increasing levels of postoperative systemic inflammation on POD3 divided into quartiles, hereafter titled “inflammation groups”, with the lowest quartile being the refence group.

**Primary endpoints: disability-free survival and quality of recovery at POD3 and POD30**

The binary primary outcome, persistent disability or death to 90 days, will be compared between inflammation groups using log-binomial regression to estimate risk ratios and 95% confidence intervals. Should the log-binomial model fail to converge, modified Poisson regression with robust standard errors will be used.

The QOR endpoints will be compared between inflammation groups using median regression.

A sensitivity analysis will adjust for RELIEF randomised group, age, sex, ASA physical status, Charlson score, preoperative aspirin, haemoglobin (baseline), type of surgery, planned HDU/ICU (either), and duration of surgery.

Methods for addressing missing POD3 CRP are provided below.

**Secondary endpoints:**

The binary secondary (S) outcomes [S1 to S4, S7] will be compared between inflammation groups using log-binomial regression to estimate risk ratios and 95% confidence intervals directly. Should the log-binomial model fail to converge, modified Poisson regression with robust standard errors will be used.

Duration of stay outcomes [S5 and S6] will be summarised using medians and interquartiles ranges, and compared across inflammation groups using parametric accelerated failure time models.

All secondary endpoints will have unadjusted and adjusted analysis undertaken. Adjusting for RELIEF randomised group, age, sex, ASA physical status, Charlson score, dexamethasone, type of surgery, planned HDU/ICU (either), duration of surgery, and blood transfusion to POD3. Further possible confounders will be determined prior to commencement of the statistical analysis by examining.

**Missing data**

Missing POD3 CRP pre-postoperative POD3 characteristics will be compared with those who have the measurement present. If there is evidence of missing CRP data being dependent on these characteristics then multiple imputation will be performed using chained equations, including baseline and post-baseline variables (up until POD3), including the relevant outcome variables in the imputation models.

**Subgroup analysis**

An exploratory subgroup analyses will be done for the inflammatory groups by RELIEF randomised groups. The endpoints persistent disability or death to 90 days, acute kidney injury, POD3 QoR-15, surgical site infection and hospital stay will be calculated for each CRP inflammation group by RELIEF randomised group. For these analyses, we will undertake tests for interaction by adding treatment-by-endpoint terms to the regression models specified for the main analyses of each outcome.

This will be reported as a web-supplement.

**Analysis of other biomarkers of inflammation**

Other markers of inflammation (preoperative white cell count (WCC), lowest postoperative albumin, highest WCC to discharge, highest temperature) and length of stay will have medians and interquartile range reported for each CRP inflammation group. Median regression will be undertaken to compare these measures between the CRP inflammation groups.

**Spline analysis**

A continuous spline analysis of CRP and QOR to POD3 and persistent disability or death to 90 days.
