# Supplementary material for: Postoperative systemic inflammation after major abdominal surgery: patient‐centred outcomes
Source: Anaesthesia. 2023 Aug 2;78(11):1365–75. doi: 10.1111/anae.16104 (PMC10952313; doi:10.1111/anae.16104)
Supplement: Supplementary file 2 — Figure S1. Direct acyclic graph outlining the relationship of plasma CRP, postoperative systemic inflammatory dysregulation, primary patient‐centred and other (secondary) outcomes. [file ANAE-78-1365-s002.docx]

**Figure S1**. A direct acyclic graph outlining the relationship of plasma CRP, postoperative systemic inflammatory dysregulation (PSID), primary patient-centred and other (secondary) outcomes. The model includes measured confounding variables, measured mediating complications (hyperinflammation and immunosuppression) and unmeasured intrinsic patient specific mediators. AKI: acute kidney injury, ASA: American Society of Anesthesiologists, CRP: C-reactive protein, DNA: deoxyribose nucleic acid, HDU: high dependency unit, ICU: intensive care unit, SSI: surgical site infection, QoR; quality of recovery.
