# Supplementary material for: Postoperative systemic inflammation after major abdominal surgery: patient‐centred outcomes
Source: Anaesthesia. 2023 Aug 2;78(11):1365–75. doi: 10.1111/anae.16104 (PMC10952313; doi:10.1111/anae.16104)
Supplement: Supplementary file 3 — Table S1. Subgroup analysis for interaction between treatment allocation and outcome in different CRP quartiles. Table S2. Values of other inflammatory markers according to CRP quartiles. Table S3. Patient characteristics by missing maximum CRP up to day 3 divided by missing status. [file ANAE-78-1365-s003.docx]

**Table S1:** Subgroup analysis for interaction between treatment allocation and outcome in different CRP quartiles

| Outcome | CRP quartile | Restrictive | Restrictive - RR^*^ (95% CI) | Liberal | Liberal - RR^*^ (95% CI) | Interaction p-value |
| --- | --- | --- | --- | --- | --- | --- |
| Disability to 90 days or death |  |  |  |  |  |  |
|  | q1 | 12.8 | Ref | 8.8 | Ref |  |
|  | q2 | 14.1 | 1.10 (0.7, 1.6) | 12.3 | 1.39 (0.9, 2.2) | 0.47 |
|  | q3 | 18.7 | 1.46 (1.0, 2.1) | 17.5 | 1.99 (1.3, 3.1) |  |
|  | q4 | 25.3 | 1.97 (1.4, 2.8) | 25.9 | 2.94 (2.0, 4.4) |  |
| AKI |  |  |  |  |  |  |
|  | q1 | 2.7 | Ref | 3.0 | Ref |  |
|  | q2 | 7.1 | 2.60 (1.2, 5.7) | 2.2 | 0.72 (0.3, 1.9) | 0.21 |
|  | q3 | 8.2 | 3.00 (1.4, 6.4) | 3.5 | 1.16 (0.5, 2.7) |  |
|  | q4 | 14.7 | 5.38 (2.6, 11.2) | 8.1 | 2.68 (1.3, 5.4) |  |
| Surgical site infection (any) confirmed |  |  |  |  |  |  |
|  | q1 | 6.8 | Ref | 5.5 | Ref |  |
|  | q2 | 12.0 | 1.77 (1.1, 2.9) | 10.6 | 1.94 (1.1, 3.4) | 0.99 |
|  | q3 | 19.3 | 2.86 (1.8, 4.5) | 16.1 | 2.94 (1.8, 4.9) |  |
|  | q4 | 28.7 | 4.24 (2.8, 6.5) | 22.9 | 4.21 (2.6, 6.9) |  |
| Hosp LoS to 30 days with deaths censored |  |  |  |  |  |  |
|  | q1 | 3.6 (2.4, 6.2) | Ref | 3.6 (2.4, 5.5) | Ref |  |
|  | q2 | 5.5 (3.4, 8.7) | 1.48 (1.3, 1.7) | 5.5 (3.5, 8.5) | 1.55 (1.4, 1.7) | 0.91 |
|  | q3 | 6.7 (4.6, 12.4) | 2.01 (1.8, 2.3) | 6.7 (4.6, 11.8) | 1.99 (1.8, 2.2) |  |
|  | q4 | 9.6 (6.3, 16.7) | 2.75 (2.4, 3.1) | 9.3 (6.4, 15.6) | 2.74 (2.4, 3.1) |  |
| Quality of recovery, day 3 |  |  |  |  |  |  |
|  | q1 | 115.3 (100.3, 128.5) | Ref | 119.0 (104.3, 129.2) | Ref |  |
|  | q2 | 108.8 (91.8, 123.2) | -6.50 (-10.3, -2.7) | 109.8 (91.7, 123.1) | -9.15 (-13.3, -5.0) | 0.72 |
|  | q3 | 102.8 (85.8, 116.3) | -12.55 (-16.5, -8.6) | 103.5 (88.7, 117.2) | -15.55 (-19.9, -11.2) |  |
|  | q4 | 96.0 (78.0, 112.5) | -19.30 (-24.1, -14.5) | 98.7 (78.5, 112.6) | -20.30 (-25.2, -15.4) |  |
| Outcomes are RRs, except for QOR which is differences in medians and LOS which are HRs. *RR is the relative risk of the outcome for each CRP quartile relative to the lowest CRP quartile. | | | | | | |

| **Table S2**: Values of other inflammatory markers according to CRP quartiles – unadjusted analysis | | | | | | | |
| --- | --- | --- | --- | --- | --- | --- | --- |
| Factor (CRP;mg.l^-1^) | q1: ≤85 | q2: >85-140 | q3: >140-200 | q4: >200-587 | CRP q2 vs q1 | CRP q3 vs q1 | CRP q4 vs q1 |
| N | 639 | 634 | 642 | 618 |  |  |  |
| Lowest albumin (g.l^-1^) to day 3 | 31.1 (28.0, 34.0) | 29.0 (26.0, 32.0) | 28.0 (25.0, 31.0) | 26.0 (23.0, 30.0) | -2.05 (-2.99, -1.11), p<0.001 | -3.05 (-3.97, -2.13), p<0.001 | -5.05 (-5.93, -4.17), p<0.001 |
| Highest WCC (10^9^.l^-1^) to discharge | 11.8 (9.7, 14.6) | 12.4 (10.2, 15.2) | 13.4 (10.6, 17.2) | 14.8 (11.7, 18.7) | 0.58 (0.05, 1.12), p=0.03 | 1.64 (1.08, 2.20), p<0.001 | 3.07 (2.50, 3.65), p<0.001 |
| Highest temperature (^o^C) to discharge | 37.3 (37.0, 37.6) | d37.5 (37.2, 37.8) | 37.5 (37.2, 37.9) | 37.7 (37.4, 38.2) | 0.20 (0.11, 0.28), p<0.001 | 0.21 (0.15, 0.26), p<0.001 | 0.44 (0.30, 0.57), p<0.001 |

WCC=white cell count, CRP=C-reactive protein

| **Table S3**: Patient characteristics by missing maximum CRP up to day 3 divided by missing status | | |
| --- | --- | --- |
| Factor | Not missing | Missing |
| N | 2533 | 450 |
| Age, mean (SD) | 66.6 (12.7) | 63.2 (12.9) |
| Male | 1326 (52.3%) | 228 (50.7%) |
| Country |  |  |
| Australia | 1422 (56.1%) | 255 (56.7%) |
| Canada | 414 (16.3%) | 83 (18.4%) |
| Other | 697 (27.5%) | 112 (24.9%) |
| Body weight — kg, median (IQR) | 82.3 (68.0, 101.2) | 87.9 (74.0, 105.0) |
| BMI kg/m2, median (IQR) | 29.3 (24.8, 35.9) | 31.2 (26.2, 37.2) |
| Preoperative WHODAS score, median (IQR) | 15.0 (13.0, 21.0) | 15.5 (13.0, 20.0) |
| Hypertension | 1526 (60.2%) | 281 (62.4%) |
| Coronary artery disease | 382 (15.1%) | 80 (17.8%) |
| Heart failure | 86 (3.4%) | 18 (4.0%) |
| Previous myocardial infarction | 217 (8.6%) | 51 (11.3%) |
| Peripheral vascular disease | 158 (6.2%) | 29 (6.4%) |
| Charlson score, median (IQR) | 2.0 (1.0, 4.0) | 2.0 (1.0, 3.0) |
| cancer2 | 1778 (70.2%) | 280 (62.2%) |
| Current smoker | 325 (12.8%) | 73 (16.2%) |
| History of stroke or TIA | 191 (7.5%) | 29 (6.4%) |
| COPD | 422 (16.7%) | 76 (16.9%) |
| Moderate / severe renal disease | 184 (7.3%) | 25 (5.6%) |
| ASA |  |  |
| ASA 1 or 2 | 982 (38.8%) | 146 (32.4%) |
| ASA 3 or 4 | 1551 (61.2%) | 304 (67.6%) |
| Aspirin | 491 (19.4%) | 89 (19.8%) |
| Preoperative steroids | 126 (5.0%) | 29 (6.4%) |
| NSAIDS | 136 (5.4%) | 31 (6.9%) |
| Baseline haemoglobin g/L, median (IQR) | 133.0 (120.0, 144.0) | 135.0 (121.0, 147.0) |
| WCC, median (IQR) | 7.3 (6.0, 9.0) | 7.6 (6.3, 9.2) |
| Albumin, median (IQR) | 38.0 (35.0, 42.0) | 38.0 (32.0, 41.0) |
| Proposed surgery |  |  |
| GI surgery | 1825 (72.0%) | 287 (63.8%) |
| Renal/urological, gynaecological or other | 708 (28.0%) | 163 (36.2%) |
| Surgical technique |  |  |
| Laparoscopic | 764 (30.2%) | 157 (34.9%) |
| Open (includes conversion from laparoscopy) | 1769 (69.8%) | 293 (65.1%) |
| Cancer surgery | 1655 (65.3%) | 255 (56.7%) |
| Duration of surgery (hours), median (IQR) | 3.3 (2.4, 4.5) | 3.2 (2.3, 4.4) |
| Planned destination ICU/HDU vs ward | 756 (29.8%) | 95 (21.1%) |
| Blood loss, median (IQR) | 200.0 (100.0, 400.0) | 200.0 (100.0, 500.0) |
| Blood transfusion pre day 3 | 275 (10.9%) | 43 (9.7%) |
| Discharge alive pre day 3 | 157 (6.2%) | 93 (20.7%) |
| RCT - Liberal fluid group | 1264 (49.9%) | 229 (50.9%) |

BMI=body mass index, TIA=transient ischemic attack, COPD=chronic obstructive pulmonary disease, ASA=American Society of Anesthesiologists, NSAIDS= non-steroidal anti-inflammatory drugs, WCC= white cell count, PACU=post-anaesthesia care unit, ICU=intensive care unit, HDU=high dependency unit, CVP=central venous pressure, Hb=haemoglobin.
